# Comparing the performance of the novel FAMCAT algorithms and established case-finding criteria for familial hypercholesterolaemia in primary care

**DOI:** 10.1136/openhrt-2021-001752

**Published:** 2021-10-11

**Authors:** Nadeem Qureshi, Ralph K Akyea, Brittany Dutton, Jo Leonardi-Bee, Steve E Humphries, Stephen Weng, Joe Kai

**Affiliations:** 1 Primary Care Stratified Medicine (PRISM) Research Group, School of Medicine, University of Nottingham, Nottingham, UK; 2 Centre for Evidence Based Healthcare, Faculty of Medicine and Health Sciences, University of Nottingham, Nottingham, UK; 3 Centre for Cardiovascular Genetics, Institute of Cardiovascular Science, University College London, London, UK; 4 Cardiovascular and Metabolism, Janssen Research & Development, High Wycombe, UK

**Keywords:** electronic health records, hyperlipidemias, delivery of health care

## Abstract

**Objective:**

Familial hypercholesterolaemia (FH) is a common inherited disorder causing premature coronary heart disease (CHD) and death. We have developed the novel Familial Hypercholesterolaemia Case Ascertainment Tool (FAMCAT 1) case-finding algorithm for application in primary care, to improve detection of FH. The performance of this algorithm was further improved by including personal history of premature CHD (FAMCAT 2 algorithm). This study has evaluated their performance, at 95% specificity, to detect genetically confirmed FH in the general population. We also compared these algorithms to established clinical case-finding criteria.

**Methods:**

Prospective validation study, in 14 general practices, recruiting participants from the general adult population with cholesterol documented. For 260 participants with available health records, we determined possible FH cases based on FAMCAT thresholds, Dutch Lipid Clinic Network (DLCN) score, Simon-Broome criteria and recommended cholesterol thresholds (total cholesterol >9.0 mmol/L if ≥30 years or >7.5 mmol/L if <30 years), using clinical data from electronic and manual extraction of patient records and family history questionnaires. The reference standard was genetic testing. We examined detection rate (DR), sensitivity and specificity for each case-finding criteria.

**Results:**

At 95% specificity, FAMCAT 1 had a DR of 27.8% (95% CI 12.5% to 50.9%) with sensitivity of 31.2% (95% CI 11.0% to 58.7%); while FAMCAT 2 had a DR of 45.8% (95% CI 27.9% to 64.9%) with sensitivity of 68.8% (95% CI 41.3% to 89.0%). DLCN score ≥6 points yielded a DR of 35.3% (95% CI 17.3% to 58.7%) and sensitivity of 37.5% (95% CI 15.2% to 64.6%). Using recommended cholesterol thresholds resulted in DR of 28.0% (95% CI 14.3% to 47.6%) with sensitivity of 43.8% (95% CI 19.8% to 70.1%). Simon-Broome criteria had lower DR 11.3% (95% CI 6.0% to 20.0%) and specificity 70.9% (95% CI 64.8% to 76.5%) but higher sensitivity of 56.3% (95% CI 29.9% to 80.2%).

**Conclusions:**

In primary care, in patients with cholesterol documented, FAMCAT 2 performs better than other case-finding criteria for detecting genetically confirmed FH, with no prior clinical review required for case finding.

**Trial registration number:**

NCT03934320.

**Key questions**

**What is already known about this subject?**

- Over 80% of people with familial hypercholesterolaemia (FH) still remain undiagnosed and untreated, but systematic searching of primary care health records can identify individuals at increased risk of FH and is recommended by national (National Institute for Health and Care Excellence, NICE) guidelines.
- The Familial Hypercholesterolaemia Case Ascertainment Tool (FAMCAT 1) algorithm, at a prespecified threshold related to prevalence of the condition, has been shown to perform well at predicting patients with FH documented in primary care databases, with the updated FAMCAT 2 algorithm performing better at predicting these FH patients than the original version.

**What does this study add?**

- The FAMCAT 2 algorithm performed better at identifying genetically confirmed FH than the original version, yielding higher levels of detection and testing less individuals who did not have FH (higher sensitivity) in primary care. The Dutch Lipid Clinic Network criteria score ≥6 points or using NICE-recommended cholesterol thresholds had lower detection rates and sensitivity than FAMCAT 2 but still performed well, while the Simon-Broome criteria for possible FH showed limited detection rate and specificity in this setting.

**How might this impact on clinical practice?**

- FAMCAT 2 provides an automated method of searching individuals’ primary care electronic health records to yield higher numbers of subsequently genetically confirmed FH than existing methods for case finding.
- Using routinely available health record data, FAMCAT 2 offers the further advantage of minimising the need for prior clinical review and examination of patients; and streamlining referrals for further specialist assessment.

## Introduction

Familial hypercholesterolaemia (FH) is an inherited cause of raised cholesterol resulting in premature coronary heart disease (CHD) and greatly increased mortality risk, when left untreated.^1–3^ In most countries, over 80% of people with FH remain undiagnosed.^4 5^ This presents a major challenge for health systems, with as many as 1 in 250 people affected.^6^ Existing international guidelines recommend clinicians use clinical tools such as Dutch Lipid Clinic Network (DLCN) score or Simon-Broome criteria to detect possible FH, prior to definitive genetic testing.^4 7 8^ However, in addition to cholesterol measures, all these tools require detailed family histories of hypercholesterolaemia and premature CHD and examination for physical signs of FH, to be undertaken.^1 9^ This makes them less useful for case-finding in the general community population. Recent UK guidelines have additionally recommended systematically identifying potential cases through searching primary care electronic healthcare records (EHRs) for patients using certain cholesterol thresholds (under 30 years old with cholesterol over 7.5 mmol/L or over 9.0 mmol/L in older patients), roughly aligned to the 99th population centile for cholesterol levels in the general population.^7^ Further consideration is that these primary care FH case-finding tools, used to identify initial possible FH cases, should have high specificity to ensure that most of those with actual FH are not missed.

Current systematic approaches to identify FH in primary care records only identify a minority of patients with FH.^10–13^ To improve detection of the majority of individuals still not diagnosed with the condition, we developed a Familial Hypercholesterolaemia Case Ascertainment Tool (FAMCAT).^14–16^ The FAMCAT algorithm takes account of the interaction between family history, statin prescribing, triglycerides and secondary causes, and focuses on those factors that are readily extractable from routine entries in patients’ EHRs, searching the available data to identify those with highest likelihood of FH. It is intended as a case-finding tool to identify those eligible for further assessment, specialist referral and genetic testing for possible FH.

The FAMCAT 1 algorithm was originally derived and internally validated using data from 3 million patients in UK primary care using the Clinical Practice Research Datalink—broadly representative of the UK general population in terms of sex, age and ethnicity. It includes elements of existing clinical criteria tools, such as DLCN and Simon-Broome, in addition to other variables such as triglyceride level, and clinical diagnosis of diabetes and chronic kidney disease based on coded records, to improve diagnostic accuracy.^14^ FAMCAT has been further externally validated in two large UK primary care population studies.^15 16^ These three previous studies showed FAMCAT was highly predictive of FH documented in primary care records with similar performance (area under the curve (AUC) between 0.83 to 0.86). A further advancement of the algorithm has been developed (FAMCAT 2) by incorporating past history of premature CHD, leading to an improved AUC of 0.87.^15^ We originally evaluated the FAMCAT 1 and FAMCAT 2 algorithms at an FH probability threshold of 0.002 which aligns to the original estimated FH prevalence of 1 in 500. This prevalence is now accepted to be 1 in 250.^6^ In this study, to optimise the clinical value of the FAMCAT 1 and 2 case-finding algorithms, we estimated the performance and probability threshold of these algorithms at 95% specificity, using FH genetic testing as the reference standard. Further, we compared the performance of the algorithms at this specificity against established case-finding criteria (DLCN, Simon-Broome and recommended cholesterol threshold) in this population.

## Methods

### Study population

Twenty-three General Practices, in the catchment areas of two East Midland’s lipid specialist services, expressed an interest to participate with 14 general practices recruited from May 2017 to November 2019, with a total practice population of 193 589. To ensure diverse study populations were recruited, five general practices predominately serving urban areas were recruited, together with four serving suburban areas and four from rural areas. The updated FH case-finding tool, incorporating FAMCAT 2, was installed on practice computers as part of a free-to-use FH audit tool in primary care^17^ and used to identify potentially eligible patients who were then offered genetic blood testing for FH.

### Participants

Participants were eligible if they were aged 18 years or over, had a serum cholesterol recorded in their EHRs, without a previous diagnosis of FH.

Participants were invited for genetic testing if identified by the practice administrator to have FAMCAT probability of FH above 0.002 (0.2%, as defined in previous studies of FAMCAT^14 15^ based on an EHRs search. For all participants with access to their EHR who underwent confirmatory genetic testing, we recalculated the FAMCAT 1 probability threshold score and calculated the FAMCAT 2 and DLCN scores, together with Simon-Broome criteria, confirming and expanding the information already collected through manual review of EHRs and further verified by a validated family history questionnaire (seeonline supplemental file 1).^18^ Use of any of these criteria, except for FAMCAT 1 and 2, for case finding is recommended in current English (National Institute for Health and Care Excellence, NICE) guidelines for best practice.^7^ The data captured included all FAMCAT variables, and data used for other clinical case finding criteria: Simon-Broome criteria variables (total and low-density lipoprotein cholesterol (LDL-C), family history of premature CHD and/or hypercholesterolaemia, clinical signs of FH), DLCN criteria variables (LDL-C, clinical history of coronary heart, cerebrovascular and peripheral vascular disease, family history, clinical signs of FH).^4^ Full details of the variables for each case-finding criteria are given in online supplemental table 1.

10.1136/openhrt-2021-001752.supp2 Supplementary data

10.1136/openhrt-2021-001752.supp1 Supplementary data

### FAMCAT algorithms

The original (FAMCAT 1) algorithm consists of nine diagnostic indicators stratified by gender.^14^ This includes total cholesterol (TC) or LDL-cholesterol, age during cholesterol measurement, triglycerides, lipid-lowering drug usage, family history of FH, family history of CHD, family history of raised cholesterol, diabetes and chronic kidney disease. Full details of variables are provided in online supplemental table 1.

A subsequent version of the algorithm (FAMCAT 2), also included coded personal history of premature CHD and re-estimated the regression equations with TC, LDL-cholesterol, triglycerides and age as continuous variables to improve its calibration.^15^ The regressions functions and equations are fully published elsewhere.^15^

### Reference standard

Confirmed FH was defined as a laboratory determined pathogenic variant (designated here as a mutation) in *LDLR*, *APOB, PCSK9* or *LDLRAP1* identified. The genetic testing was conducted by the Bristol National Health Service (NHS) Genetics Laboratory, using a next-generation sequencing assay covering all coding exons and intron–exon boundaries of LDLR, APOB and PCSK9 plus a assay for copy number variants for LDLR insertions/deletions. The report outputs also identified variants of unknown significance (VUS). The laboratory staff conducting genetic testing were blinded to the FAMCAT scores, cholesterol levels, DLCN score and Simon-Broome classification of the participants.

### Patient and public involvement

Our patient and public study coinvestigator was involved in the design and conduct of this study. The patient representatives participated in interpreting the study results and assisted with the plain English summary.

### Statistical analysis

We compared the characteristics of male and female recruited participants. The detection rate of genetically confirmed FH at optimal probability thresholds were estimated for the FAMCAT 1 and FAMCAT 2 algorithms, Simon-Broome criteria for possible FH, DLCN score ≥6 and recommended TC thresholds alone (TC >9.0 mmol/L in those 30 years +; TC >7.5 in those under 30 years, approximating to the 99th centile for the general population). The optimal threshold was defined as that achieved at a specificity of 95% (ie, only missing 1 in 20 patients with FH). The analysis was also performed at a specificity of 90% specificity. The probability threshold is the cut-off where high risk of FH is defined and was varied from 0.002 to 0.25. Further the sensitivity, specificity, positive predictive value (PPV) and negative predictive value (NPV), and the AUC were calculated. For all performance metrics, 95% CIs were calculated based on Wilson score interval method.^19^ PPV and NPV were calculated based on the study prevalence of genetically confirmed FH. All analyses were conducted using STATA V.16.1.

The study was registered with clinical trial.gov (NCT03934320).

## Results

### Study process

**Figure 1:**
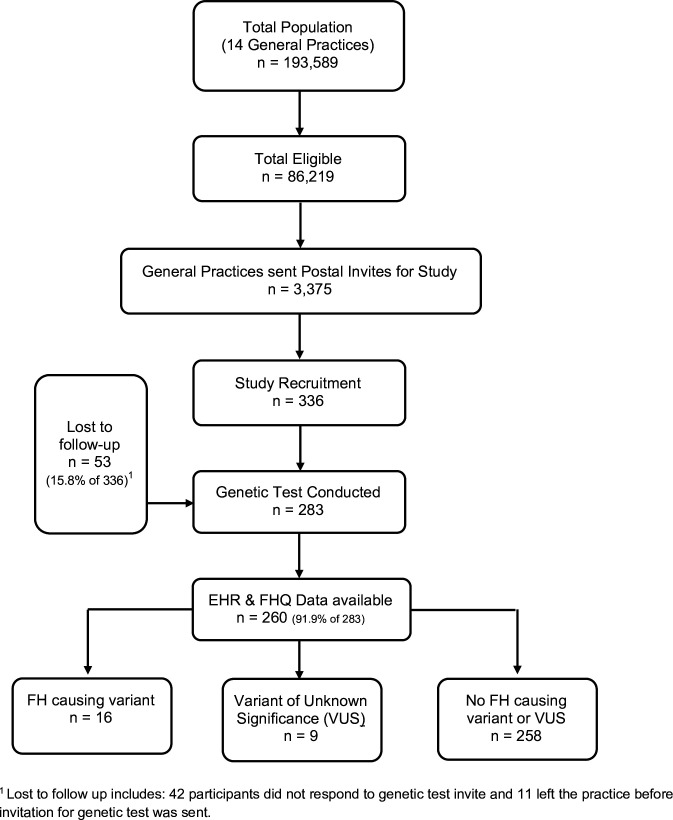
Study flow diagram. EHR, Electronic Health Records; FH, familial hypercholesterolaemia; FHQ, family history questionnaire; VUS, variant of unknown significance.

From a total adult population of 193 589 patients from 14 general practices, 86 219 (44.4%) had a TC or LDL-C assessed and documented in their EHRs. Full clinical data from the EHR was available for 260 study participants (figure 1), of which 16 participants were identified with a confirmed FH mutation (13 *LDLR,* 3 *APOB,* 1 *PCSK9*), 10 participants were identified with a VUS, while 234 participants (90%) did not have a known FH pathogenic mutation or documented as VUS. These VUS results were reviewed by three experts and they confirmed none were consistent with known FH pathogenic mutations.

### Characteristics of recruited participants

The average age of the 260 recruited participants was 57.5 years, 80% self-reported as white British and 69% were women. Twenty-five per cent of participants had a statin prescribed and 50% had a family history of premature CHD in first-degree relatives. As demonstrated in table 1, women were older and had lower FAMCAT probability scores.

**Table 1:** Characteristics of the recruited participants

Characteristics	Men (n=80)	Women (n=180)	P value
Age, years* median (IQR)	53.0 (46.0–59.0)	59.0 (51.5–66.5)	<0.0001
Highest total cholesterol, mmol/L median (IQR)	7.4 (6.6–8.0)	7.7 (6.8–8.3)	0.023
Median highest Low-density lipoprotein cholesterol, mmol/L median (IQR)	5.0 (4.0–5.3)	5.1 (4.3–5.7)	0.047
Family history of premature coronary heart disease†	41 (51.3)	89 (49.4)	0.788
Statin prescribed	24 (30%)	42 (23%)	0.254
FAMCAT 1 probability of FH, median (IQR)	0.047 (0.20–0.091)	0.010 (0.003–0.217)	<0.0001
Fulfils Simon-Broome possible FH criteria‡	21 (26.3%)	59 (32.8%)	0.293

P value, Mann-Whitney U test for continuous variables and χ^2^ test for categorical variables.

*Age at time of recruitment into the study.

†Premature defined as coronary heart disease <50 years in second-degree relative or <60 years in first-degree relative.

‡Simon-Broome possible FH criteria defined as having a total cholesterol >7.5 mmol/L or LDL-C >4.9 mmol/L and a family history of premature coronary heart disease.

FAMCAT, Familial Hypercholesterolaemia Case Ascertainment Tool; FH, familial hypercholesterolaemia.

### Performance of FAMCAT 1 and FAMCAT 2 algorithms

**Figure 2:**
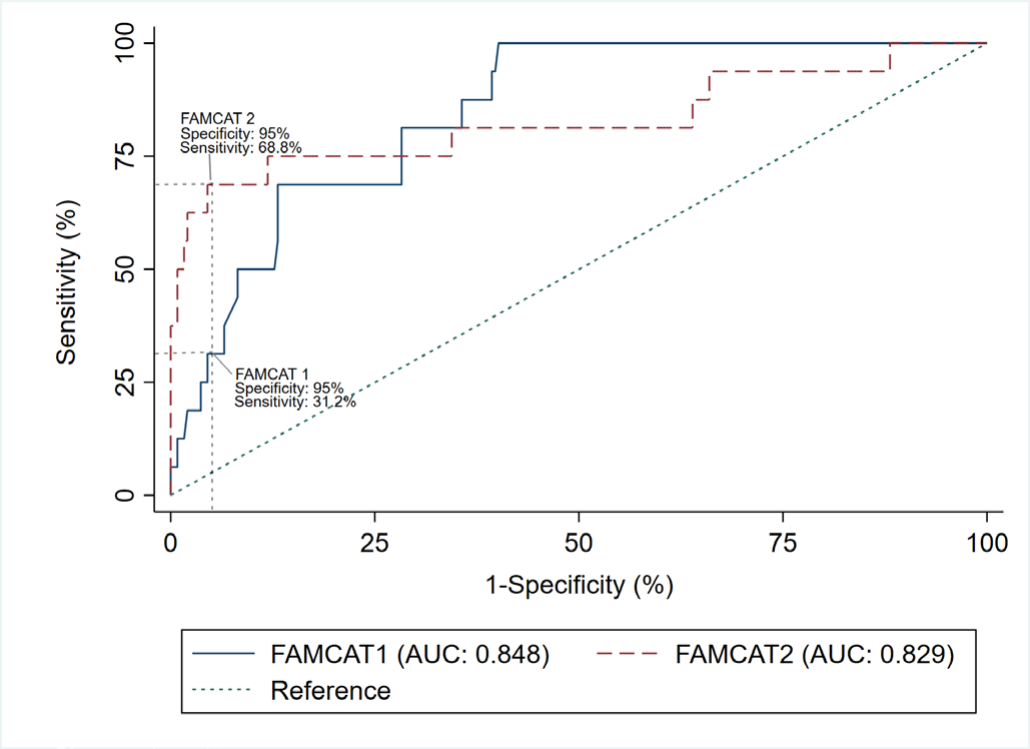
Receiver operating characteristic curve and sensitivity of FAMCAT 1 and FAMCAT 2 algorithms at 95% specificity (n=260). AUC, area under the curve; FAMCAT, Familial Hypercholesterolaemia Case Ascertainment Tool.

At 95% specificity, the FAMCAT 1 algorithm achieved a detection rate of 27.8% (95% CI 12.5% to 50.9%) and sensitivity of 31.2% (95% CI 11.0% to 58.7%), with an FH probability threshold of 0.140 (figure 2). With a specificity of 90%, the detection rate dropped slightly to 25.0% (95% CI 13.3% to 42.1%) but sensitivity increased to 50.0% (95% CI 24.7% to 75.3%), with a probability threshold of 0.080. In the case of the FAMCAT 2 algorithm, at 95% specificity the detection rate was 45.8% (95% CI 27.9% to 64.9%) and sensitivity of 68.8% (95% CI 41.3% to 89.0%), with a probability threshold of 0.005 (figure 2). With a specificity of 90%, the detection rate dropped to 30.6% (95% CI 18.0% to 46.9%) with identical sensitivity 68.8% (95% CI 41.3% to 89.0%), with a probability threshold of 0.004. Full details on all metrics at the different thresholds of the FAMCAT 1 and FAMCAT 2 is presented in online supplemental table 2.

From the 260 participants completing genetic testing, and health records available, 32 participants had FAMCAT probability scores below the original study selection criteria (probability at or above 0.002). Online supplemental table 3 and online supplemental figure 1 present the full performance metrics when these 32 participants were excluded. When limited to 228 participants, at 95% specificity for FAMCAT 1 algorithm, the detection rate was 28.6% (95% CI 11.7% to 54.7%) and sensitivity 25.0% (95% CI 7.3% to 52.4%)), with a similar probability threshold of 0.160 and the FAMCAT 2 algorithm had similar detection rate (50.0% (95% CI 29.9% to 70.1%)), sensitivity 62.5% (95% CI 35.4% to 84.8%) and probability threshold (0.005).

### Comparison to other case-finding tools

By applying English NICE recommendations for case-finding to the genetic tested individuals, the performance of Simon-Broome, DLCN and cholesterol thresholds could be assessed and compared with FAMCAT 1 and 2 (table 2). The Simon-Broome criteria had a substantially lower detection rate of 11.3% (95% CI 6.0% to 20.0%), only identifying 9 cases from 80 fulfilling the criteria, with a sensitivity of 56.3% (95% CI 29.9% to 80.2%) and specificity of 70.9% (95% CI 64.8% to 76.5%). The DLCN ≥6 points had a higher detection rate of 35.3% (95% CI 17.3% to 58.7%) and specificity of 95.5% (95% CI 92.1% to 97.7%) but lower sensitivity (37.5%, 95% CI 15.2% to 64.6%); and the recommended cholesterol thresholds had a similar detection rate of 28.0% (95% CI 14.3% to 47.6%) with sensitivity of 43.8% (95% CI 19.8% to 70.1%) and a specificity of 92.6% (95% CI 88.6% to 95.6%). PPV ranged from 10.3% (Simon-Broome) to 43.4% (FAMCAT 2), with a high NPV maintained of at least 96%.

**Table 2:** NICE recommended criteria performance (detection rate, sensitivity, specificity, PPV, NPV, AUC) for identifying monogenic FH (n=260)

Threshold for the various case-finding tools	FH positive above threshold	FH positive below threshold	Detection rate	Sensitivity (95% CI)	Specificity (95% CI)	PPV* (95% CI)	NPV* (95% CI)	AUC (95% CI)
FAMCAT 1 (at 0.140 threshold)	5/18	11/242	27.8% (12.5%–50.9%)	31.2% (11.0% to 58.7%)	94.7% (91.1% to 97.1%)	25.8% (12.8% to 45.2%)	95.9% (94.4% to 97.0%)	0.63 (0.51 to 0.75)
FAMCAT 2 (at 0.0047 threshold)	11/24	5/236	45.8% (27.9%–64.9%)	68.8% (41.3% to 89.0%)	94.7% (91.1% to 97.1%)	43.4% (28.3% to 57.4%)	98.1% (96.1% to 99.0%)	0.82 (0.70 to 0.94)
Simon-Broome Possible FH (TC >7.5 mmol/L or low-density lipoprotein cholesterol >4.9 mmol/L and family history premature CHD^+^)	9/80	7/180	11.3% (6.0%–20.0%)	56.3% (29.9% to 80.2%)	70.9% (64.8% to 76.5%)	10.3% (6.7% to 15.3%)	96.5% (94.0% to 97.9%)	0.64 (0.51 to 0.76)
Dutch Lipid Clinical Network Criteria Score ≥6 points (Probable FH)	6/17	10/243	35.3% (17.3%–58.7%)	37.5% (15.2% to 64.6%)	95.5% (92.1% to 97.7%)	33.0% (17.5% to 52.5%)	96.3% (94.7% to 97.4%)	0.66 (0.54 to 0.79)
TC >9.0 mmol/L (≥30 years) OR TC >7.5 mmol/L (<30 years)	7/25	9/235	28.0% (14.3%–47.6%)	43.8% (19.8% to 70.1%)	92.6% (88.6% to 95.6%)	26.0% (14.8% to 40.9%)	96.5% (94.8% to 97.7%)	0.68 (0.56 to 0.81)

Detection rate.

*PPV and NPV assumes a prevalence of FH of 0.056 based on study.

†Premature defined as coronary heart disease <50 years in second-degree relative or <60 years in first-degree relative.

†95% CI calculated using the Wilson score interval discussed in Brown, LD, Cat, TT and DasGupta, A (2001). Interval Estimation for a proportion. Statistical Science 16:101–133.

AUC, area under the curve; FAMCAT, Familial Hypercholesterolaemia Case Ascertainment Tool; FH, familial hypercholesterolaemia; NICE, National Institute for Health and Care Excellence; NPV, negative predictive value; PPV, positive predictive value; TC, total cholesterol.

## Discussion

### Principal findings

By comparing the performance of the FAMCAT algorithms against the reference standard of an FH genetic test, we identified that the FAMCAT 2 algorithm performed better than the original version, yielding higher levels of detection and sensitivity. Also, comparisons against the DLCN criteria score ≥6 points or using the NICE recommended cholesterol thresholds had lower detection rates and sensitivity than FAMCAT 2 but still performed well, while the Simon-Broome criteria for possible FH showed limited detection rate and specificity in this setting.

### Comparison with existing studies

This is the first study, internationally, to evaluate and compare case-finding approaches for FH in the primary care population, confirmed with genetic diagnosis as the primary outcome. Existing evidence on case finding in the community has been limited to using clinical phenotype as the FH primary outcome of interest.^10 11 13 20^ As would be expected, when identifying genetically confirmed FH in primary care, there was lower detection rates than elicited in specialist settings, such as lipid clinics, where genetic detection rates of 20%–38% is achieved using the possible Simon-Broome criteria and up to 41% detection in those with DLCN scores greater than or equal to 6.^21–23^ Further, electronic case finding for FH using DLCN criteria requires specific clinical findings to have been recorded (tendon xanthoma, premature arcus cornealis) to complete the assessment, which is uncommon in primary care.^4 24^ Previous studies in primary care have thus required FH nurse specialists to identify these clinical signs.^25 26^

### Strengths and limitations

This study has several strengths. The FAMCAT algorithm has been validated against genetic testing in a national diagnostic laboratory. The research has also been pragmatically undertaken in a routine primary care setting. General practitioners were left to identify patients eligible for genetic testing for FH using the FAMCAT algorithm. This supports the fidelity of the approach, and its generalisability to usual clinical practice. In contrast, many commonly used clinical algorithms for risk stratification and prediction have not been validated in their intended settings, being solely based on retrospective modelling before their adoption in guidelines for practice.^27 28^

The genetic testing used is gold-standard diagnostic confirmation of FH conducted by accredited English NHS laboratories. We only classified patients with FH if they had a clearly identified pathogenic variant for FH in their laboratory results. The capture of clinically coded data from patient electronic records, confirmation of family history using a validated questionnaire, and further manual record extraction in practice allowed for triangulation and improved validity of the clinical variables used in the case-finding criteria. This ensured accurate comparisons between FH case-finding criteria, including Simon-Broome and DLCN criteria, and cholesterol thresholds.^1 4 7^ The inclusion of patients below the initial 0.002 thresholds for all of the five criteria enabled better estimates of specificity and NPV for each case-finding criteria.

We recognise some study limitations. In the study, patients had to have a prior cholesterol measurement to be included in the study, so the recruitment strategy would have missed those who may have FH but never had a cholesterol measurement. Further, our study participants expectedly had overall higher cholesterol levels than the general primary care population. However, in real-world setting, the starting point for any FH case-finding tool will usually be those patients with serum cholesterol recorded in their primary care EHRs with a focus on those with higher cholesterol concentrations. Recognising patients 40 years and over would have had cholesterol testing as part of the national cardiovascular disease (CVD) health check programme, while those under 40 are unlikely to be measured unless clinical indicated, a subgroup analysis stratified at 40 would have been informative but too few participants with cholesterols below 40 years (n=23) were available to enable a meaningful analysis. Also, in this study, we collated comprehensive family histories leading to a significant proportion of patients with a family history of premature CHD being identified. This highlights the importance of improved family history recording in primary care and the benefits of targeted case-finding and assessment to those patients at greatest risk.

### Clinical implications

The current failure to diagnose the majority of people with FH leads to either no treatment or inadequate treatment of people wrongly thought to have commoner ‘life-style related’ elevated cholesterol. As a result, patients with undetected FH will suffer avoidable premature CVD and higher risk of early death. FAMCAT provides an automated method of searching patients’ EHRs to subsequently yield higher numbers of genetically confirmed FH than existing methods for case finding. Using routinely available health record data, FAMCAT offers the further advantage of not requiring the collection of new information or prior clinical review and examination of patients. It can thus be used as a case-finding tool to more efficiently identify adults with possible FH in the community and streamline referrals for further specialist assessment and genetic testing.^29^ This novel approach appears superior to case-finding using the Simon-Broome criteria, which was developed from historical secondary care data. This should be coordinated with other models for case-finding, cascade testing of relatives and child screening.^4 7 9 28^

To optimise FH identification in primary care, the initial FH case finding tool should maximise specificity, to avoid missing FH patients. Therefore, the performance of the FAMCAT 1 and FAMCAT 2 was assessed at specificity of 90% and 95%. If healthcare resources are limited, probability of FH thresholds could be altered to improve the FH cases detection rate and reduce numbers sent for genetic testing but this would be at the expense of missing FH patients. This study also supports this decision making, by providing details of the sensitivity and specificity at different FAMCAT probability thresholds. With global interest in primary prevention of CVD, increasing numbers of adults will have cholesterol measurements, increasing the opportunity to apply the FAMCAT algorithm in EHRs. Fundamentally, FAMCAT and other primary care case-finding criteria need the cholesterol measurement available and this cannot be imputed into the algorithm.^30^ Also the primary care EHRs need to be kept up-to-date with key variables, such as, the family history.^31 32^ Those patients with a family history of premature CHD should have this information coded in their records and recalled for cholesterol testing. Further research on the implementation of FAMCAT in practice is now warranted.

## Conclusions

We have demonstrated that in primary care, in patients with raised cholesterol levels documented in medical records, this new case-finding algorithm (FAMCAT) performs better than DLCN score, Simon-Broome criteria or cholesterol thresholds alone for detecting genetically confirmed FH, with no prior clinical review or examination required for case finding.

## Data Availability

No data are available. We do not have consent from participants to share their data for the purposes of future research.
