# Supplementary material for: Comparing the performance of the novel FAMCAT algorithms and established case-finding criteria for familial hypercholesterolaemia in primary care
Source: Open Heart. 2021 Oct 11;8(2):e001752. doi: 10.1136/openhrt-2021-001752 (PMC8506870; doi:10.1136/openhrt-2021-001752)
Supplement: Supplementary data [file openhrt-2021-001752supp002.pdf]

Participant number (for research purposes): | | | | | | | | | | | | | | | |

The University of Nottingham
UNITED KINGDOM · CHINA · MALAYSIA

GENETIC RISK OF HEART DISEASE QUESTIONNAIRE

Please complete this questionnaire about you and your family's health. The information you give will be looked at by the doctors. You will be advised if the doctor suggests any further enquiries or investigations, based on this information.

Name……

Date of Birth ……………………....……. (DD/MM/YYYY)

Please tick the boxes that best describe your parents' ethnic group:

	Father	*Mother*
White British or Irish (or other North European)	☐	☐
Mediterranean (includes Cypriot, Italian, Turkish, Kurdish etc.)	☐	☐
European of Jewish Origin	☐	☐
Black (includes Caribbean, African, other Black)	☐	☐
Indian continent (Indian, Pakistani, Bangladeshi, East African Asian, Sri Lankan)	☐	☐
Chinese (or other South East Asian)	☐	☐
Middle East (includes Iranian, Iraqi, etc.)	☐	☐
Other *(Please write your answer)*________________________	☐	☐
Not known	☐	☐

Have you ever had any significant medical illnesses or operations since birth?

Name of illness/ Type of operation	Age when diagnosed or at time of operation
1.	
2.	
3.	
4.	
5.	

© The University of Nottingham 2017. All rights reserved. Applications for the copyright owner's permission to modify any part of this publication should be forwarded in writing to Professor Nadeem Qureshi, Primary Care, School of Medicine, Queen's Medical Centre, Derby Road, Nottingham NG7 2U

Qureshi N, *et al. Open Heart* 2021; 8:e001752. doi: 10.1136/openhrt-2021-001752

Participant number (for research purposes): | | | | | | | | | | | | | | | |

(Blank page)

© The University of Nottingham 2017. All rights reserved. Applications for the copyright owner's permission to modify any part of this publication should be forwarded in writing to Professor Nadeem Qureshi, Primary Care, School of Medicine, Queen's Medical Centre, Derby Road, Nottingham NG7 2U

Participant number (for research purposes): | | | | | | | | | | | | | | | |

**This section asks about your IMMEDIATE RELATIVES**	**Do not give information about family members who are not your blood relatives** e.g. step- or foster- brothers or sisters or children/ relatives by marriage

Relationship to you	First name (optional)	Approximate age, or year of birth	Any history of **RAISED CHOLESTEROL** *Please state Yes or No*	Any history of **HEART ATTACK** or **MYOCARDIAL INFARCTION (MI)** *Please state Yes or No*	Age when **first HEART ATTACK** or **MI** occurred	Any history of **ANGINA** *Please state Yes or No*	If this person died, what was their age?	What was the cause of death?	Tick (✓) if unknown
**Your mother**									
**Your father**									
**Your grandparents**									
Your mother's mother									
Your mother's father									
Your father's mother									
Your father's father									

© The University of Nottingham 2017. All rights reserved. Applications for the copyright owner's permission to modify any part of this publication should be forwarded in writing to Professor Nadeem Qureshi, Primary Care, School of Medicine, Queen's Medical Centre, Derby Road, Nottingham NG7 2U

Participant number (for research purposes): ☐☐☐☐☐☐☐☐☐☐☐☐☐☐☐

How many of the following blood relatives do you have (including any who have died)?

Please put the number in the box. If you are unsure of the exact number, please put an approximate number.

.

		On your mother's side		On your father's side	
**Brothers** (include half brothers)	☐	**Uncles**:	☐	**Uncles**:	☐
**Sisters** (include half sisters)	☐	**Aunts**:	☐	**Aunts**:	☐
**Sons** *	☐				
**Daughters** *	☐				

* Complete this only for sons and daughters for whom you are the biological parent.

You do not need to give any more information about living relatives, *unless* they have a history of raised cholesterol, have had heart disease or heart operations

Please give details on the following sheets for relatives

- who have had heart attack, also known as Myocardial Infarction (MI)
- who have, or have had, angina
- who have died

© The University of Nottingham 2017. All rights reserved. Applications for the copyright owner's permission to modify any part of this publication should be forwarded in writing to Professor Nadeem Qureshi, Primary Care, School of Medicine, Queen's Medical Centre, Derby Road, Nottingham NG7 2U

Participant number (for research purposes):

This section asks about illnesses in your BROTHERS and SISTERS

Relationship to you	Put **M** for mother's side, **F** for father's side	First name (optional)	Approximate age, or year of birth	Any history of **RAISED CHOLESTEROL** *Please state Yes or No*	Any history of **HEART ATTACK** or **MYOCARDIAL INFARCTION (MI)** *Please state Yes or No*	Age when **first HEART ATTACK** or **MI** occurred	Any history of **ANGINA** *Please state Yes or No*	If this person died, what was their age?	What was the cause of death?
**Your sisters or half**									
**Your brothers or half brothers**									

Please use the last sheet of this questionnaire if you require more space for your answers about brothers and sisters

© The University of Nottingham 2017. All rights reserved. Applications for the copyright owner's permission to modify any part of this publication should be forwarded in writing to Professor Nadeem Qureshi, Primary Care, School of Medicine, Queen's Medical Centre, Derby Road, Nottingham NG7 2U

Participant number (for research purposes): | | | | | | | | | | | | | | | |

This section asks about illnesses in more distant BLOOD RELATIVES

Relationship to you	Put **M** for mother's side, **F** for father's side	First name (optional)	Approximate age, or year of birth	Any history of **RAISED CHOLESTEROL** *Please state Yes or No*	Any history of **HEART ATTACK** or **MYOCARDIAL INFARCTION (MI)** *Please state Yes or No*	Age when **first HEART ATTACK** or **MI** occurred	Any history of **ANGINA** *Please state Yes or No*	If this person died, what was their age?	What was the cause of death?
**Your Uncles**									
**Your Aunts**									

Please use the last sheet of this questionnaire if you require more space for your answers about uncles and aunts

© The University of Nottingham 2017. All rights reserved. Applications for the copyright owner's permission to modify any part of this publication should be forwarded in writing to Professor Nadeem Qureshi, Primary Care, School of Medicine, Queen's Medical Centre, Derby Road, Nottingham NG7 2U

Participant number (for research purposes): | | | | | | | | | | | | | | | |

This section asks about illnesses in your sons and daughters

Relationship to you	First name (optional)	Approximate age, or year of birth	Any history of **RAISED CHOLESTEROL** *Please state Yes or No*	Any history of **HEART ATTACK** or **MYOCARDIAL INFARCTION (MI)** *Please state Yes or No*	Age when **first HEART ATTACK** or **MI** occurred	Any history of **ANGINA** *Please state Yes or No*	If this person died, what was their age?	What was the cause of death?
**Your sons**								
**Your daughters**								

Please use the last sheet of this questionnaire if you require more space for your answers about other relatives

© The University of Nottingham 2017. All rights reserved. Applications for the copyright owner’s permission to modify any part of this publication should be forwarded in writing to Professor Nadeem Qureshi, Primary Care, School of Medicine, Queen’s Medical Centre, Derby Road, Nottingham NG7 2U

Participant number (for research purposes): | | | | | | | | | | | | | | | |

Information about illnesses in other relatives

**Relationship to you**	Put **M** for mother's side, **F** for father's side	First name (optional)	Approximate age, or year of birth	Any history of **RAISED CHOLESTEROL** *Please state Yes or No*	Any history of **HEART ATTACK** or **MYOCARDIAL INFARCTION (MI)** *Please state Yes or No*	Age when **first HEART ATTACK** or **MI** occurred	Any history of **ANGINA** *Please state Yes or No*	If this person died, what was their age?	What was the cause of death?

PLEASE USE THIS SPACE BELOW IF YOU REQUIRE MORE SPACE FOR ANY OF YOUR ANSWERS OR TO GIVE ANY COMMENTS

Thank you for completing this questionnaire. Please return it in the enclosed freepost envelope with your consent form, contact details and Protecting Your Heart Questionnaire.

© The University of Nottingham 2017. All rights reserved. Applications for the copyright owner's permission to modify any part of this publication should be forwarded in writing to Professor Nadeem Qureshi, Primary Care, School of Medicine, Queen's Medical Centre, Derby Road, Nottingham NG7 2U
