# Supplementary material for: Comparing the performance of the novel FAMCAT algorithms and established case-finding criteria for familial hypercholesterolaemia in primary care
Source: Open Heart. 2021 Oct 11;8(2):e001752. doi: 10.1136/openhrt-2021-001752 (PMC8506870; doi:10.1136/openhrt-2021-001752)

Supplementary Material

Comparing novel and established case-finding criteria for familial hypercholesterolaemia in primary care

Qureshi N, *et al. Open Heart* 2021; 8:e001752. doi: 10.1136/openhrt-2021-001752

Supplementary Table 1: Availability of standard diagnostic criteria variables in manual extraction from EHR

	Score	Number (%)
**Dutch Lipid Criteria Network (DLCN)**		
Family History		
First-degree relative with premature coronary and/or vascular disease (men ≤ 55 years, women ≤ 60 year)	1	130 (50%)
First-degree relative with known LDL-C ≥ 95th percentile for age and sex	1	-
Clinical history		
Patient with premature coronary artery disease (men ≤ 55 years, women ≤ 60 years)	2	22 (8.5)
Patient with premature cerebral or peripheral vascular disease (men ≤ 55 years, women ≤ 60 years)	1	4 (1.5)
**Physical examination**		
Tendon xanthomata	6	0
Arcus cornealis at age ≤ 45 years	4	0
**LDL cholesterol level (mmol/L)**		
≥ 8.5	8	4 (1.5)
6.5 – 8.4	5	14 (5.4)
5.0 – 6.4	3	121 (46.5)
4.0 – 4.9	1	59 (22.7)
**Simon Broome Diagnostic criteria**		
Family History		
Family history of myocardial infarction: aged younger than 50 years in second-degree relative or aged younger than 60 years in first-degree relative.	-	130 (50)
Family history of raised total cholesterol: greater than 7.5 mmol/l in adult first- or second-degree relative)	-	37 (14.2)
Clinical history		
Patient with premature coronary artery disease (men ≤ 55 years, women ≤ 60 years)	-	22 (8.5)
**Physical examination**		
Tendon xanthomata	-	0
**Cholesterol level**		
Total cholesterol > 7.5mmol/l or LDL-Cholesterol > 4.5 mmol/l	-	171 (65.8)

Qureshi N, *et al. Open Heart* 2021; 8:e001752. doi: 10.1136/openhrt-2021-001752

Continuation of Supplementary Table 1…

	Score	Number (%)
**FAMCAT 1 algorithm**		
Highest total or LDL cholesterol (mmol/L)	-	
Ideal (TC ≤ 5 or LDL ≤ 3.3)		27 (10.4)
High (TC>5 to ≤6.5 OR LDL>3.3 to ≤4.1)		30 (11.5)
Very High (TC>6.5 to ≤7.5 OR LDL>4.1 to ≤4.9)		53 (20.4)
Extremely High (TC>7.5 OR LDL>4.9)		150 (57.7)
Age during cholesterol measurement (years)	-	
16-24		3 (1.2)
25-34		12 (4.6)
35-44		40 (15.4)
45-54		75 (28.9)
55-64		75 (28.9)
65-74		47 (18.1)
75-84		8 (3.1)
85 or above		0
Triglycerides (mmol/L)	-	
Ideal (<1.7)		127 (48.9)
Borderline high (≥1.7 to<2.3)		51 (19.6)
High (≥2.3 to<5.6)		68 (26.2)
Very high (≥5.6)		7 (2.7)
Not Recorded		7 (2.7)
Treatment with lipid lowering drugs during cholesterol measurement	-	
No lipid lowering drugs prescribed		194 (74.6)
Low potency statins		2 (0.8)
Medium potency statin		47 (18.1)
High potency statins		17 (6.5)
Family history of myocardial infarction	-	131 (50.4)
Family history of raised cholesterol	-	37 (14.2)
Family history of familial hypercholesterolaemia	-	9 (3.5)
Any diagnosis of diabetes	-	16 (6.2)
Any diagnosis of kidney disease	-	7 (2.7)
**FAMCAT 2 algorithm**		
Highest LDL cholesterol ever (mmol/L), median (IQR)	-	5.0 (4.2 – 5.6)
Highest total cholesterol ever (mmol/L), median (IQR)		7.6 (6.7 – 8.2)
Age during cholesterol measurement (years), mean (SD)	-	53.9 (12.1)
Triglycerides (mmol/L), mean (SD)	-	1.7 (1.3 – 2.5)
Previous history of premature myocardial infarction	-	22 (8.5)

Qureshi N, *et al. Open Heart* 2021; 8:e001752. doi: 10.1136/openhrt-2021-001752

Supplementary Table 2. **FAMCAT performance** (detection rate, sensitivity, specificity, positive and negative predictive values, AUC) for identifying genetically-confirmed familial hypercholesterolaemia at pre-specified probability thresholds (***n=260***)

Threshold	FH Positive Above Threshold	FH Positive Below Threshold	Detection rate	Sensitivity (95% CI)	Specificity (95% CI)	PPV* (95% CI)	NPV* (95% CI)	AUC (95% CI)
**FAMCAT 1**								
0.002	16/228	0/32	7.0% (4.4 – 11.1%)	100%	13.1% (0.1 – 18.0%)	6.4% (5.7 – 6.8%)	100%	0.57 (0.54 – 0.59)
0.004	16/200	0/60	8.0% (5.0 – 12.6%)	100%	24.6% (19.3 – 30.5%)	7.3% (6.8 – 7.8%)	100%	0.63 (0.60 – 0.65)
0.010	16/159	0/101	10.1% (6.3 – 15.7%)	100%	41.4% (35.1 – 47.9%)	9.2% (8.4 – 10.1%)	100%	0.71 (0.68 – 0.74)
0.050	11/54	5/206	20.4% (11.8 – 32.9%)	68.8% (41.3 – 89.0%)	82.4% (77.0 – 86.9%)	18.8% (12.9 – 25.7%)	97.8% (95.6 – 98.8%)	0.76 (0.64 – 0.88)
0.080	8/32	8/228	25.0% (13.3 – 42.1%)	50.0% (24.7 – 75.3%)	90.2% (85.7 – 93.6%)	23.2 (13.9 – 35.2%)	96.8% (95.0 – 98.0%)	0.70 (0.57 – 0.83)
0.100	6/22	10/238	27.3% (13.2 – 48.2%)	37.5% (15.2 – 64.6%)	93.4% (89.6 – 96.2%)	25.3% (13.6 – 41.9%)	96.2% (94.6 – 97.4%)	0.65 (0.53 – 0.78)
0.140	5/18	11/242	27.8% (12.5 – 50.9%)	31.3% (11.0 – 58.7%)	94.7% (91.1 – 97.1%)	25.8% (12.8 – 45.2%)	95.9% (94.4 – 97.0%)	0.63 (0.51 – 0.75)
0.150	5/16	11/244	31.3% (14.2 – 55.6%)	31.3% (11.0 – 58.7%)	95.5% (92.1 – 97.7%)	29.1% (14.4 – 49.9%)	95.9% (94.5 – 97.1%)	0.63 (0.52 – 0.75)
0.200	3/9	13/251	33.3% (12.1 – 64.6%)	18.8% (4.0 – 45.6%)	97.5% (94.7 – 99.1%)	31.1% (12.1 – 60.5%)	95.3% (94.2 – 96.3%)	0.58 (0.48 – 0.68)
0.250	2/5	14/255	40.0% (11.8 – 76.9%)	12.5% (1.6 – -38.3%)	98.8% (96.4 – 99.7%)	37.6% (11.5 – 74.1%)	95.0% (94.1 – 96.0%)	0.56 (0.47 – 0.64)
**FAMCAT 2**								
0.002	12/79	4/181	15.2% (8.9 – 24.7%)	75.0% (47.6 – 92.7%)	72.5% (66.5 – 78.0%)	13.9% (10.0 – 18.3%)	98.0% (95.4 – 99.0%)	0.74 (0.62 – 0.85)
0.0036	11/36	5/224	30.6% (18.0 – 46.9%)	68.6% (41.3 – 89.0%)	89.8% (85.2 – 93.3%)	28.5% (19.1 – 38.7%)	98.0% (95.9 – 98.9%)	0.79 (0.67 – 0.91)
0.004	11/29	5/231	37.9% (22.7 – 56.0%)	68.8% (41.3 – 89.0%)	92.6% (88.6 – 95.6%)	35.6% (23.5 – 47.9%)	98.0% (96.0 – 99.0%)	0.81 (0.69 – 0.93)
0.0047	11/24	5/236	45.8% (27.9 – 64.9%)	68.8% (41.3 – 89.0%)	94.7% (91.1 – 97.1%)	43.4% (28.3 – 57.4%)	98.1% (96.1 – 99.0%)	0.82 (0.70 – 0.94)
0.010	8/10	9/250	80.0% (49.0 – 94.3%)	50.0% (24.7 – 75.3%)	99.2% (97.1 – 99.9%)	78.3% (43.7 – 91.6%)	97.1% (95.4 – 98.2%)	0.75 (0.62 – 0.87)
0.050	3/3	13/257	100%	18.8% (4.0 – 45.6%)	100%	100%	95.4% (94.3 – 96.4%)	0.59 (0.49 – 0.69)
0.100	2/2	14/258	100%	12.5% (1.6 – 38.3%)	100%	100%	95.1% (94.2 – 96.0%)	0.56 (0.48 – 0.65)

* Positive predictive value (PPV) and negative predictive value (NPV) assumes a prevalence of familial hypercholesterolaemia (FH) of 0.056 based on study.

Qureshi N, *et al. Open Heart* 2021; 8:e001752. doi: 10.1136/openhrt-2021-001752

AUC – area under the curve; CI – confidence interval; FH – familial hypercholesterolaemia; NPV – negative predictive value; PPV – positive predictive value.

Detection rate - 95% confidence interval calculated using the Wilson score interval discussed in Brown, LD, Cat, TT and DasGupta, A (2001). Interval Estimation for a proportion. Statistical Science 16:101-133.

Supplementary Table 3. **FAMCAT performance** (detection rate, sensitivity, specificity, positive and negative predictive values, AUC) for identifying genetically-confirmed familial hypercholesterolaemia at pre-specified probability thresholds (***n=228***)

Threshold	FH Positive Above Threshold	FH Positive Below Threshold	Detection rate	Sensitivity (95% CI)	Specificity (95% CI)	PPV* (95% CI)	NPV* (95% CI)	AUC (95% CI)
**FAMCAT 1**								
0.004	16/200	0/28	8% (5.0 – 12.6%)	100%	13.2% (9.0 – 18.5%)	6.4% (5.7 – 6.8%)	100%	0.57 (0.54 – 0.59)
0.010	16/159	0/69	10.1% (6.3 – 15.7%)	100%	32.5% (26.3 – 39.3%)	8.1% (7.0 – 8.8%)	100%	0.66 (0.63 – 0.69)
0.050	11/54	5/174	20.4% (11.8 – 32.9%)	68.8% (41.3 – 89.0%)	79.7% (73.7 – 84.9%)	16.7% (11.4 – 23.1%)	97.7% (95.4 – 98.8%)	0.74 (0.62 – 0.86)
0.085	8/30	8/198	26.6% (14.2 – 44.5%)	50.0% (24.7 – 75.3%)	89.6% (84.7 – 93.4%)	22.2% (13.2 – 34.2%)	96.8% (94.9 – 98.0%)	0.70 (0.57 – 0.83)
0.100	6/22	10/206	27.3% (13.2 – 48.2%)	37.5% (15.2 – 64.4%)	92.5% (88.0 – 95.6%)	22.8% (12.0 – 38.5%)	96.1% (94.5 – 97.3%)	0.65 (0.53 – 0.77)
0.160	4/14	12/214	28.6% (11.7 – 54.7%)	25.0% (7.3 – 52.4%)	95.3% (91.5 – 97.7%)	23.9% (10.6 – 46.1%)	95.5% (94.2 – 96.7%)	0.60 (0.49 – 0.71)
0.150	5/16	11/212	31.3% (14.2 – 55.6%)	31.3% (11.0 – 58.7%)	94.8% (90.9 – 97.4%)	26.3% (12.8 – 46.3%)	95.9% (94.4 – 97.0%)	0.63 (0.51 – 0.75)
0.200	3/9	13/219	33.3% (12.1 – 64.6%)	18.8% (4.0 – 45.6%)	97.2% (93.9 – 99.0%)	28.2% (10.7 – 57.1%)	95.3% (94.2 – 96.3%)	0.58 (0.48 – 0.68)
0.250	2/5	14/223	40.0% (11.8 – 76.9%)	12.5% (1.6 – 38.3%)	98.6% (95.9 – 99.7%)	34.4% (10.2 – 71.4%)	95.0% (94.1 – 96.0%)	0.56 (0.47 – 0.64)
**FAMCAT 2**								
0.002	12/78	4/150	15.4% (9.0 – 25.0%)	75.0% (47.6 – 92.7%)	68.9% (62.2 – 75.0%)	12.5% (9.0 – 16.5%)	97.9% (95.2 – 99.0%)	0.72 (0.61 – 0.83)
0.0038	11/33	5/195	33.3% (19.8 – 50.4%)	68.6% (41.3 – 89.0%)	89.6% (84.7 – 93.4%)	28.2% (18.6 – 38.8%)	98.0% (95.9 – 98.9%)	0.79 (0.67 – 0.91)
0.004	11/28	5/200	39.3% (23.6 – 57.6%)	68.8% (41.3 – 89.0%)	92.0% (87.5 – 95.3%)	33.7% (21.9 – 46.0%)	98.0% (96.0 – 99.0%)	0.80 (0.68 – 0.92)
0.0047	10/20	6/208	50.0% (29.9 – 70.1%)	62.5% (35.4 – 84.8%)	95.3% (91.5 – 97.7%)	44.0% (27.0 – 59.9%)	97.7% (95.8 – 98.7%)	0.79 (0.67 – 0.91)
0.010	8/10	8/218	80.0% (49.0 – 94.3%)	50.0% (24.7 – 75.3%)	99.1% (96.6 – 99.9%)	75.9% (40.3 – 90.4%)	97.1% (95.4 – 98.2%)	0.75 (0.62 – 0.87)
0.050	3/3	13/225	100%	18.8% (4.0 – 45.6%)	100%	100%	95.4% (94.3 – 96.4%)	0.59 (0.49 – 0.69)
0.100	2/2	14/226	100%	12.5% (1.6 – 38.3%)	100%	100%	95.1% (94.2 – 96.0%)	0.56 (0.48 – 0.65)

* Positive predictive value (PPV) and negative predictive value (NPV) assumes a prevalence of familial hypercholesterolaemia (FH) of 0.056 based on study.

AUC – area under the curve; CI – confidence interval; FH – familial hypercholesterolaemia; NPV – negative predictive value; PPV – positive predictive value.

Qureshi N, *et al. Open Heart* 2021; 8:e001752. doi: 10.1136/openhrt-2021-001752

Detection rate - 95% confidence interval calculated using the Wilson score interval discussed in Brown, LD, Cat, TT and DasGupta, A (2001). Interval Estimation for a proportion. Statistical Science 16:101-133.

Supplementary Figure 1: ROC curve and sensitivity of FAMCAT 1 & FAMCAT2 algorithms at 95% specificity (n=228)

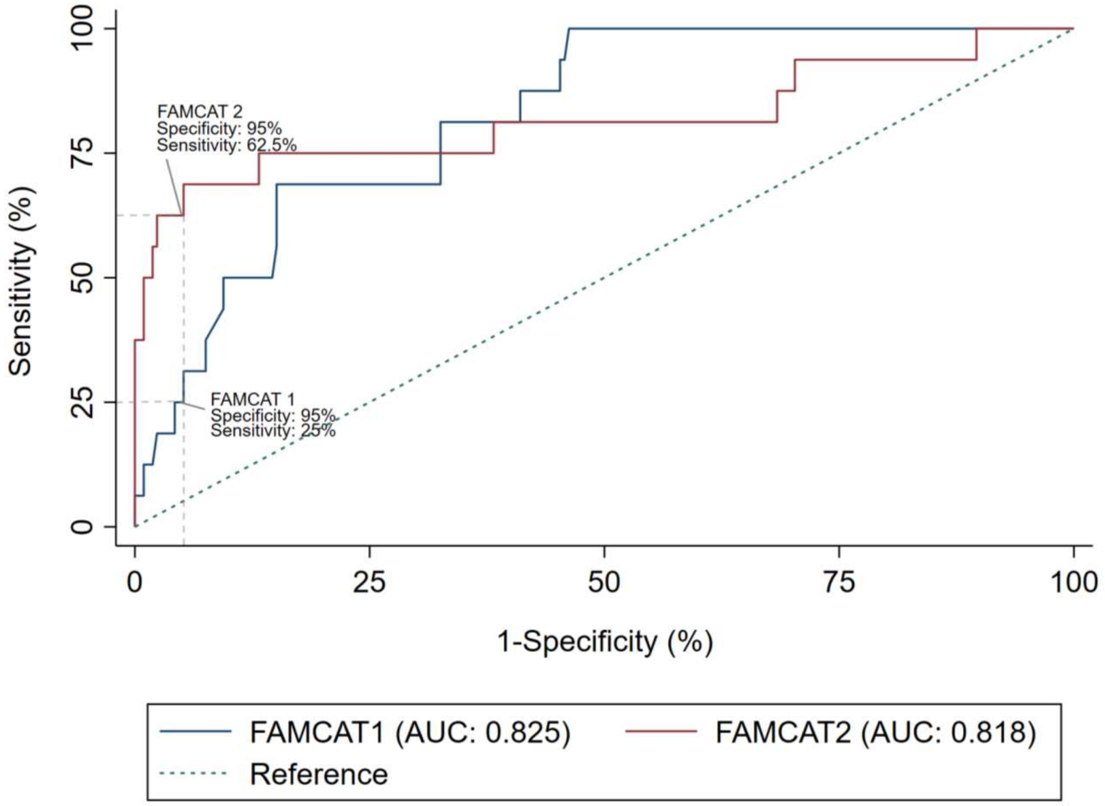

Supplement: Supplementary data [file openhrt-2021-001752supp001.pdf]
